# Methodology and Applications of Disease Biomarker Identification in Human Serum

**Published:** 2007-02-14

**Authors:** Ziad J. Sahab, Suzan M. Semaan, Qing-Xiang Amy Sang

**Affiliations:** Department of Chemistry and Biochemistry and Institute of Molecular Biophysics, Florida State University, Tallahassee, FL 32306-4390, U.S.A

**Keywords:** Serum biomarkers, proteomics, cancer signatures, disease diagnosis and prognosis, biochemical indicators, technological innovations

## Abstract

Biomarkers are biomolecules that serve as indicators of biological and pathological processes, or physiological and pharmacological responses to a drug treatment. Because of the high abundance of albumin and heterogeneity of plasma lipoproteins and glycoproteins, biomarkers are difficult to identify in human serum. Due to the clinical significance the identification of disease biomarkers in serum holds great promise for personalized medicine, especially for disease diagnosis and prognosis. This review summarizes some common and emerging proteomics techniques utilized in the separation of serum samples and identification of disease signatures. The practical application of each protein separation or identification technique is analyzed using specific examples. Biomarkers of cancers of prostate, breast, ovary, and lung in human serum have been reviewed, as well as those of heart disease, arthritis, asthma, and cystic fibrosis. Despite the advancement of technology few biomarkers have been approved by the Food and Drug Administration for disease diagnosis and prognosis due to the complexity of structure and function of protein biomarkers and lack of high sensitivity, specificity, and reproducibility for those putative biomarkers. The combination of different types of technologies and statistical analysis may provide more effective methods to identify and validate new disease biomarkers in blood.

## 1. Introduction

### 1.1 Proteomics

Proteomics is the science and technology of separating and identifying proteins from crude biological samples. It is mostly significant when differentially-expressed proteins between two samples that belong or that are subject to different conditions are identified. This identification will allow for the characterization of biological roles, clarification of biological mechanisms, and identification of therapeutic targets and biomarkers. The first step of a proteomic study requires almost always the separation of proteins using electrophoresis or chromatography techniques followed by the identification steps that are performed using mass spectrometry. The latter measures accurately the masses of peptides generated from the digestion of the protein by trypsin or another enzyme followed by the use of software that scan the different protein databases available to identify the protein. This identification is based on the information collected about this protein throughout the study including but not limited to isoelectric point, molecular mass, peptide masses, and specie of the biological sample. It is preferable–even required sometimes–to use immunoblotting techniques or N-terminal sequencing to validate the identity of the proteins. Proteomics studies can be applied on any protein mixture extracted from any organism including plant, bacterial, and animal cells. Proteomics strategies have been used to identify disease-specific protein markers called biomarkers that could provide the basis for the development of new diagnosis methodologies, treatments, and early disease detection (Hanash, 2000; Somiari et al. 2003).

### 1.2 Clinical proteomics and biomarkers

The term clinical proteomics refers to “the application of available proteomics technologies to current areas of clinical investigation” (Clarke et al. 2003). Many diseases manifest themselves through severe changes in human physiology, which forms the basis for clinical chemistry and bestows its value in diagnoses and subsequent therapeutic interventions (Bischoff and Luider, 2004). Clinical proteomics includes the global analysis of proteins expressed by the genome of an organism, with the typical aim being the evaluation of quantitative changes that occur as a function of disease, treatment, or environment (Somiari et al. 2003). Proteomics strategies have been used to recognize disease-specific protein markers called biomarkers that could provide the basis for the development of new diagnosis methodologies, treatments, and early disease detection (Hanash, 2000; Somiari et al. 2003). A biomarker is defined as “a characteristic that is objectively measured and evaluated as an indicator of normal biological processes, pathological processes, or pharmacological responses to a therapeutic intervention” (Atkinson et al. 2001).

The degree to which biomarkers reflect clinical outcomes judges their effectiveness. As biomarkers have different characteristics there should be statistical requirements that determine the usefulness of biomarkers as evaluations of disease progression or outcomes in clinical trials (Cummings, 2005). Those requirements should include statistical dispersion, detailed information on target populations, and specificity of the biomarker. Generally, an ideal biomarker is expected to be: able to detect a fundamental feature of a specific disease; validated in and confirmed by those specific disease cases; precise, able to detect the early stages of this specific disease and distinguish it from other similar disease cases or family members of that disease; simple to perform; reliable; non-invasive; and inexpensive if possible (Anon et al. 1998; Gao et al. 2005).

To identify new biomarkers of a certain disease *in vitro*, a three component analytical strategy was developed, which consists of (I) a cell line as the pre-clinical model, (II) a set of five well-studied drugs, three of which had been found in humans to elicit that disease, and (III) implementation of multi-dimensional protein identification technology (MudPIT) (see 3.3) to perform semi-quantitative analysis and identify protein biomarker candidates (Gao et al. 2005). MudPIT was chosen due to the demonstration of its usefulness in the identification as well as semi-quantification (relative changes/differential trends in protein abundance) of large numbers of proteins, both *in vivo* and *in vitro* (Washburn et al. 2001; Pang et al. 2002; Gao et al. 2003; Gao et al. 2004). This biomarker-identification stage generates a large list of biomarkers. Upon passing through a crucial second stage, a rate determining one, only the most appropriate subset of biomarker-candidates will be further tested by traditional immunoassays to identify and verify an ideal biomarker or the most credible biomarker for a specific disease according to aforementioned criteria (Gao et al. 2005).

### 1.3 Significance of proteomics of human samples

Mammalian samples are the most studied species in terms of protein profiling studies. *In vitro* culture of mammalian cell lines is an important resource for research, and have been used for disease-related studies (Tedeschi et al. 2005; Liu et al. 2005; Bianchi et al. 2005; An et al. 2005) as well as technology related ones (Hamler et al. 2004; Zhu et al. 2004; Buchanan et al. 2005). The protein profiles of cancerous cell lines have been compared to the profiles of normal cell lines (O’Neill et al. 2003). While tumor-derived cell lines can be useful for initial studies (Wu et al. 2002), each line displays a unique evolution that may not truly mimic real *in vivo* conditions (Ornstein et al. 2000a). A comparison between human prostate cell lines with tumor cells from prostate patients showed significant altered protein profiles (Ornstein et al. 2000b). Differentially-expressed proteins identified in human *in vivo* cancerous tissues when compared to their normal counterparts are by far more significant than *in vitro* ones. A large number of studies have been successful in identifying protein signatures of a disease or a condition from *in vivo* tissues (Wulfkuhle et al. 2002; Tribl et al. 2005), as well as patient serum samples (Broeckaert et al. 2000; Hathaway et al. 2005; Ahn et al. 2005). These differentially-expressed proteins are considered either the cause or the effect of the physiological change in the organism. Many published proteomic studies of human tumor tissue are associated with weaknesses in tumor representation, sample contamination by non-tumor cells and serum proteins. Studies often include a moderate number of tumors which may not be representative of clinical materials (Alaiya et al. 2005).

### 1.4 Human serum

Human serum is the clear portion of the human’s body fluid that separates from blood upon clotting. This clear fluid provides moisture to the serous membranes in the human body. It contains 60–80 mg/mL of proteins in addition to various small molecules including amino acids, lipids, salts, and sugars (Burtis et al. 2001). Normal human serum mainly contains the following proteins: IgG, IgM (Ekdahl et al. 1994), IgA, haptoglobulin, albumin (Era et al. 1995), transferrin (Burtis et al. 2001), α_1_-anti-trypsin, fibrinogen, α_2_-Macroglobulin, and complement C_3_, those account for >95% of total serum proteins (Anderson and Anderson, 2002), many of which are synthesized and secreted, shed, or lost from cells and tissues throughout the body (Schrader and Schulz-Knappe, 2001; Kennedy, 2001).

Analysis of the human serum proteome, especially for biomarkers, has great potential for diagnosis and early detection of human disease. One of the difficulties to identifying a specific marker in the human serum is the low abundance of proteins secreted in the serum as a result of the disease when compared to the high concentration of albumin, resulting from daily synthesis of ~12 g in the liver and a half-life of about 21 days (McFarlane et al. 2000), that constitutes more than 50% (w/w) of the total amount of proteins in the serum. Knowing the complexity of the human proteome and the broad dynamic range in abundance of individual proteins (e.g. albumin, immunoglobulin), there is a need for sample treatment prior to biomarker identification and is feasible using different analytical techniques. A prefractionation step to eliminate albumin from the serum is therefore required prior to the proteomic study (Lee et al. 2006a).

### 1.5 Protein properties

Proteins are composed of covalently bound amino acids. There are 20 different amino acids generating an infinite number of possible proteins. Several protein physical properties should be taken into consideration for a successful proteomic project. These properties are used to separate and to identify proteins from crude biological samples. Based on the side chain properties, amino acids of a protein can form unstable interactions through electrostatic, hydrogen bonding, and hydrophobic affinities. These interactions result in the folding of proteins that hinders their separation and identification necessitating the introduction of several reagents to the protein solubilizing buffer to break these interactions. Once these interactions are neutralized, the protein becomes unfolded (denatured). After denaturing the proteins, the remaining physical properties are used to separate and purify these proteins.

#### 1.5.1 Isoelectric point (pI)

Proteins are amphoteric molecules: they can carry positive, negative or a neutral charge. At a certain pH, the number of positive charges is equal to the number of negative charges: this pH is equal to the pI of the protein. The amino acids that affect the value of the protein isoelectric point the most are those with ionizable side chains: Arginine, Tyrosine, Lysine, Cysteine, Histidine, Glutamic acid, and Aspartic acid. Proteins can be separated based on their isolelectric point using ion exchange chromatography as well as immobilized pH gradient polyacrylamide gels (see 2.3.1).

#### 1.5.2 Hydrophobicity

The highly hydrophobic amino acid side chains are those of: Valine, Leucine, Isoleucine, Methionine, Phenylalanine, Tryptophan, and Cysteine. Proteins can be separated based on their hydrophobicities using Reversed-Phased High performance Liquid Chromatography (RP-HPLC). The stationary phase used for RP-HPLC is composed of silica beads with carbon chains linked to it. The protein mixture is injected in the column and then an acetonitrile gradient is imposed. More hydrophobic proteins require higher concentrations of acetonitrile for their elution (see 2.2.2).

#### 1.5.3 Molecular mass (M_r_) and size

Proteins can be composed of as few as tens of amino acids and as many as thousands. Most of the time, denatured proteins having a higher number of amino acids will have a bigger size and a higher M_r_. Sodium Dodecyl Sulfate Polyacrylamide gel electrophoresis (SDS-PAGE) (see 2.3.1) as well as size exclusion chromatography can be used to separate proteins based on their size or M_r_. The approximate M_r_ can be measured using these techniques. To measure the exact M_r_ of proteins a mass spectrometer should be used.

## 2. Serum Protein Separation

### 2.1 Sample treatment using commercially-available kits

The presence of highly abundant albumin and immunoglobulins that constitute approximately 60–97% of the total serum proteins gave the urge for using special kits to eliminate those highly abundant proteins to allow the visualization of low abundant proteins, such as using Cibacron blue F3GA based-resin (Ahmed et al. 2005) and antibody-related methods.

Unfortunately, it was shown that Cibacron blue F3GA based-resin method will clear, not just the albumin, but also every protein having the same pI and those with a high affinity to Cibacron Blue dye’s planar ring structure (Ahmed et al. 2003) or due to the protein’s dinucleotide fold (Lollo et al. 1999). The major disadvantage of using antibody affinity methods is the loss of the low-abundance proteins bound to high-abundance carrier proteins; this loss was reported to be up to 38 proteins in one study (Yocum et al. 2005).

### 2.2 Alternative analytical techniques for serum treatments

#### 2.2.1 Ion exchange chromatography

Ion exchange chromatography is a high resolution and capacity, easily used technique for separating proteins according to their charge. As proteins are charged molecules, adding them in a column will cause an interaction with the resin displacing mobile counter ions that are bound to the resin depending on the overall charge from the amino acids in the protein, on the pH of the buffering solution, and on the distribution of charged molecules on the folded protein’s surface. If the pH is above a protein’s pI, the protein will bind to anion exchangers (e.g. Diethylaminoethyl); below its pI it will bind to cation exchangers (e.g. Carboxy-methyl). At its pI, a protein will not bind to either a cationic or an anionic exchanger. So by using either a cation or an anion exchanger and selecting the appropriate pH, the target protein will bind to the beads in the process of purifying this protein. The typical elution modes in Ion exchange chromatography are the salt gradient (Shan and Anderson, 2002; Winnik, 2005) or the change of the eluent pH (Shan and Anderson, 2002; Alaiya et al. 2005). Ion exchange chromatography was reported to be utilized as a fractionating technique (Qin et al. 2005; Sahab Ziad et al. 2005) and as an analysis method for serum proteins (Pappas et al. 2004).

#### 2.2.2 Reversed-phase Liquid Chromatography

High performance reversed phase liquid chromatography (RPLC) is a widely accepted easy to use technique for the separation of peptides, proteins and other biopolymers (Regnier, 1983). It has great resolving power and there is ease of concentrating collected fractions, as many RPLC separations employ volatile eluents. The mobile phase, polar aqueous-organic mixtures such as acetonitrile-water or methanol-water (solvent), in RPLC is significantly more polar then the stationary phase (Anon, 2000), non-polar hydrocarbons, waxy liquids or bonded hydrocarbons (such as C_4_, C_8_, C_18_, and the like). The interaction of the non-polar components of the solutes (mixture of components = Experimented Sample) and the non-polar stationary phase cause the retention of the target component on the matrix, while the elution of the target molecule is achieved by utilizing a buffer (another solvent) of decreasing polarity. Most commonly used sorbents in RPLC are packed with chemically bonded octadecylsilyl coated silica, to which various functionalities such as the alkyl (C_18_ and C_8_), aromatic phenyl, and cyano and amino groups are widely bound. Other popular bonded phase columns have cyanopropyl, octasilyl, or phenylsilyl packings.

To separate large numbers of proteins with sufficient resolution, a 1.5 μm non porous silica-RPLC column is used. The Non-porous silica allows rapid separations of large numbers of proteins with high recovery compared to porous columns (Lubman et al. 2002). This separation is compatible with the chromatofocusing (also known as anion exchange chromatography) separation. The samples that would be separated by non porous silica reversed phase HPLC are the fractions collected from the first chromatofocusing separation. These fractions’ pH will be neutralized using HCl or NaOH, vacuum centrifuged, their precipitates will be reconstituted in water, 0.1% Trifluoroacetic acid, and 0.05% Octylglucopyranoside, and afterward will be subjected to Electrospray Mass spectrometry.

RPLC is used to fractionate serum proteins (Morris et al. 2004; Marshall et al. 2004; Qin et al. 2005; Martosella et al. 2005; Sheng et al. 2006). This method allows for a high protein recovery and is compatible with other separation techniques and can be coupled with mass spectrometry (see 2.3.2).

#### 2.2.3 Size Exclusion Chromatography

Size exclusion chromatography (SEC) is a general, not a high-resolution, technique that separates mixtures based on the molecular size of the components, such as biomolecules and organic polymers and was used as one of the several means of fractionating serum proteins (Pieper et al. 2003; Kuhn et al. 2004). Adjustment of the porosity of the gel can exclude all molecules above a certain size, therefore, the solute retention time depends sensitively on the solute’s size (Bloustine et al. 2003). A line of porous silica-based and polymer-based SEC matrices is tailored for fast, high resolution of large and small biomolecules and organic polymers. The trade names for gels available commercially in a broad range of porosities are Sephadex and Sepharose (Adrados et al. 2001). For the separation of biomolecules in aqueous systems, SEC is referred to as gel filtration chromatography, while the separation of organic polymers in non-aqueous systems is known as gel permeation chromatography.

Investigation of the binding of Human Neutrophil Peptides -1, -2 and -3 to high mass plasma proteins was performed using SEC. These serum peptides may serve as blood markers for colon cancer in combination with Surface Enhanced Laser Desorption/Ionisation—Time Of Flight/Mass spectrometry (SELDI-TOF/MS) (Albrethsen et al. 2005). This strategy may also be used for other smaller markers. The advantages and disadvantages of methodologies of serum sample treatment and prefractionation are summarized in Table 1.

### 2.3 Protein separation after sample treatment

#### 2.3.1 Two-dimensional Polyacrylamide Gel Electrophoresis (2-DE)

Two-dimensional polyacrylamide gel electrophoresis (2-DE) is an analytical technique that simultaneously separates thousands of proteins (Anderson and Anderson, 1977; Lilley et al. 2002) and allows comparative protein profiling in different crude biological samples. Although labor intensive, this technique is still the predominant method for protein profiling (Pandey and Mann, 2000; Westbrook et al. 2001; Huang et al. 2005).

A protein placed in a medium with a pH gradient and subjected to an electric field will move toward the electrode of opposite charge and stops when it arrives at the point in the pH gradient equal to its pI (Tribl et al. 2005; Sheng et al. 2006). Immobilized pH Gradient (IPG) strips offer the advantage of gradient stability over extended focusing runs, where its gradient’s pH is related with sets of acrylamido buffers that can form almost any conceivable pH gradient (Bjellqvist et al. 1982). Proteins resolved in IPG strips in the first dimension will be applied to second-dimension gels and separated by molecular mass perpendicularly to the first dimension (O’Farrell, 1975). The pores of the second dimension gel sieve proteins according to size because dodecyl sulfate coats all proteins essentially in proportion to their mass. The net effect is that proteins migrate as ellipsoids with a uniform negative charge-to-mass ratio, with mobility related logarithmically to mass. Proteins will therefore be separated horizontally based on their isoelectric point and vertically based on their molecular mass (M_r_).

Although imperfect, 2-DE is the only analytical technique that simultaneously separates thousands of proteins and allows comparative protein profiling between different crude biological samples, for instance, proteins in hepatocellular carcinoma serum have been characterized by utilizing 2-DE separation (Lee et al. 2006b). 2-DE is incapable of detecting the majority of protein components, such as very large or small proteins, membrane-associated proteins, extremely hydrophobic, acidic or basic proteins and proteins found in low abundance within the cell (Corthals et al. 2000; Gygi et al. 2000a; Gygi et al. 2000b; Graves and Haystead, 2002; Gorg et al. 2004; Chignard and Beretta, 2004). The ones that are detected are mainly the high abundant ones (Dos Remedios et al. 2003). The limitation in protein loading on the IPG strips as well as the magnitude of protein abundance within a cell that differ by up to 10 orders of magnitude are the main cause for this discrimination.

#### 2.3.2 Two-dimensional Liquid Chromatography Mass Spectrometry (2DLC-MS)

Profiling of proteins using 2-DLC can be performed (Lubman et al. 2002) using ion exchange chromatography or chromatofocusing (Whitelegge, 2005) to separate proteins based on their charge and Reversed-Phase Liquid Chromatography- Electrospray Ionization Mass spectrometer to separate proteins based on their hydrophobicities in the second dimension and measure their exact M_r_. For instance, the application of 2-DLC was used: as an isolation strategy (Lam et al. 2005), to generate protein profiles (Liao et al. 2004), and for proteomic analysis (Hamler et al. 2004) for proteins in serum.

More recently in proteomics, aiming for the optimization of protein separation methods and selective depletion of the higher abundance proteins, such as immunoglobulins (e.g. IgG) and albumin, has led researchers to separate intact proteins in the liquid phase with multidimensional liquid chromatography (MDLC). MDLC is performed utilizing separation first by chromatographic focusing and second by reversed phase, and followed by mass spectrometric detection and identification (Wall et al. 2000; Gygi et al. 2002; Pieper et al. 2003; Rose et al. 2004; Fung et al. 2004; Sheng et al. 2006). Finally, database is searched by the SEQUEST algorithm. This whole procedure is referred to as multidimensional protein identification technology (MudPIT) that will be discussed later in the review (Washburn et al. 2001) (see 3.3). In proteomics studies, MDLC successfully complements 2-D electrophoresis as it overcomes difficulties encountered during analysis of samples containing proteins spreading over a large molecular weight range (Washburn et al. 2001; Han et al. 2001) or proteins with immoderate isoelectric points (pI).

#### 2.3.3 Surface-enhanced Laser Desorption Ionization (SELDI)

Another versatile and convenient proteomics tool that allows semi quantitative analysis of numerous proteins in most biological samples is the surface-enhanced laser desorption ionization-time of flight (SELDI-TOF) system. Complex biological samples can be analyzed and compared to detect differentially-expressed proteins in the samples that are selectively retained by using different chromatographic surfaces (Tang et al. 2004), “chip” (a thin strip of aluminum, which has eight small places for loading samples), such as immobilized metal affinity capture for capturing metal-binding proteins, hydrophobic for reversed-phase capture, anion and cation exchange surfaced, and pre-activated surfaces to examine receptor-ligand, antibody-antigen, DNA-protein, and the like. The number of proteins quantified in each sample is dependent on the tissue and the chip surface. Using MS, proteins retained on the array get analyzed generating a profile of the analyzed proteome. This instrument has a combination of software, Pattern Recognition Software and one for comparing groups of biological samples, which permits the detection of subtle differences between groups of samples allowing the detection and assessment of sensitivity/specificity of a very wide range of biomarkers. An example of known software used is the Ciphergen Biosystems software (Vermeulen et al. 2005). For data presentation, the two useful formats are the grey-scale and the raw spectrum (the former represents a stained one-dimensional electrophoresis gel, hence the name “gel-view”).

Currently SELDI have been used for evaluating serum finding it useful for the discovery and identification of potential biomarkers (Li et al. 2002; Petricoin et al. 2002a; Qu et al. 2002; Petricoin et al. 2002b; Petricoin et al. 2002c; Issaq et al. 2002; Miguet et al. 2006). Although limitations during large profiling experiments, where some observations noted that spectra can vary based on analytical factors (such as the time of processing, issues in the reproducibility of SELDI (Petricoin et al. 2002a; Deng et al. 2003; Diamandis, 2003), and usually unidentified individual proteins (Chignard and Beretta, 2004)), there are advantages that include prior to analysis removal of components (salts or detergents) that commonly cause problems with other analytical tools, thus leaving only those proteins that are actively interacting with the spot surfaces to be analyzed in the ProteinChip Reader (Romer et al. 2002).

## 3. Serum Protein Identification

### 3.1 Mass spectrometry techniques

#### 3.1.1 Matrix-Assisted Laser Desorption-Ionization Time-of-Flight Mass Spectrometry (MALDI-TOF-MS): Peptide mass measurement

Proteins are usually identified utilizing Matrix-Assisted Laser Desorption-Ionization Time-of-Flight Mass Spectrometry (MALDI-TOF-MS) (Binz et al. 2003; Aebersold and Mann, 2003; Ferguson and Smith, 2003; Heck and Krijgsveld, 2004; Feuerstein et al. 2005). After 2-DE, spots are excised and treated with TPCK-treated trypsin. While in case of using 2-DLC, the resulting fractions collected in the second dimension of the separation are vacuum dried and incubated with 0.5 μg/mL TPCK-treated trypsin. The peptides generated from the trypsin digestion are first mixed with a matrix (α-cyano-hydroxycinnamic acid) (matrix application described by (Karas and Hillenkamp, 1988; Tanaka et al. 1988)), which should consist of a 100–1000-fold molar excess of the matrix over the analyte (Schiller et al. 1999). This mixture is then spotted onto a plate and allowed to evaporate forming crystals that will be analyzed by the mass spectrometer and time-of-flight analyzer. This analysis provides for measurement of the peptide fragment masses generated by trypsin digestion of the protein where the mass resolution is a measure of a spectrometer’s capability to produce separate signals of ions of similar mass (Hillenkamp et al. 1991). Finally, having obtained the molecular weight, pI, and peptide fingerprints of the protein of interest, identification of the protein should be possible using the Peptident tool that scans the Swiss-Prot protein database (UniProtKB) for possible matches.

MALDI-TOF-MS has been used to analyze serum biomarkers. For instance, it was used in combination with porous silicon nanovial arrays to achieve high-speed biomarker identification (Finnskog et al. 2006). In another study, 4 protein spots significantly down-regulated in grade 3 ovarian cancer patients were identified as isoforms of transferrin precursor using MALDI-TOF (Ahmed et al. 2005). Recently, its combination with derivatized cellulose particles represented a simple, economical, and rapid approach to generate serum protein profiles for biomarker identification (Feuerstein et al. 2005). Furthermore, one study proved that the analysis of MALDI-TOF/MS data using proteomic spectral pattern recognition software derived a distinctive molecular signature of multiple sclerosis (Avasarala et al. 2005).

#### 3.1.2 Electrospray Ionization Tandem Mass Spectrometry (ESI-MS/MS)

Electrospray ionization (Dole et al. 1968) tandem mass spectrometry (Yamashita and Fenn, 1984) (ESI-MS/MS) is a process in which a solution containing the analyte, either from a syringe pump or as the eluent flow from liquid chromatography (in this case known as LC-ESI-MS/MS), is sprayed across a high potential difference electrospray needle. The charged droplets from the spray are desolvated by either countercurrent gas flow (present in the opposite direction to the spray) or by passage through a heated capillary, a source sampling cone; then the resulting ions after desolvation of these charged droplets are analyzed in a mass spectrometer (Dongre et al. 1997). This is a very soft method of ionization where very little residual energy is retained by the analyte. This shows the importance of ESI-MS as a technique in biological studies where the analyst often requires that non-covalent molecule-protein or protein-protein interactions are representatively transferred into the gas-phase. The fact that the production of fragmentation is very little, usually none is produced, is the major disadvantage of this technique. For structural clarification studies, the requirement for tandem mass spectrometry is a must where the analyte molecules can be fragmented. ESI-MS/MS, for instance, was coupled to microcapillary reversed-phase liquid chromatography in a study where the analysis of low molecular weight serum proteome sample resulted in the identification of over 340 human serum proteins (Tirumalai et al. 2003).

The powerfulness of mass spectrometry has been discussed above. The application of mass spectrometry for the identification of disease biomarkers in serum samples is a growing field of research holding high promise for clinical oncology. Serum biomarker proteomics continues to evolve and progress following the improvements of technology, clinical study design, and bioinformatics (Drake et al. 2006).

### 3.2 Immunological techniques

#### 3.2.1 Sandwich Enzyme-Linked ImmunoSorbent Assay

A protein identification method is the “Sandwich” Enzyme-Linked ImmunoSorbent Assay (ELISA), a fast and accurate biochemical technique very useful to detect the presence and determine the concentration of low-concentrated antigen or antigen contained in high concentrations of contaminating protein in unknown samples. The sandwich ELISA requires two antibodies that bind to epitopes (antigenic sites) that do not have common ground on the antigen. This can be accomplished with either using two monoclonal antibodies that recognize discrete sites, which allow fine detection and quantification of small differences in antigen, or using one batch of affinity-purified polyclonal antibodies to pull down as much of the antigen as possible. To quantify the assay one should use a colorimetric substrate to measure the amount of labeled detection antibody bound to the matrix. As the antigen does not need to be purified prior to use and these assays are very specific, these facts are considered major advantages of this technique. However, not all antibodies can be used. Monoclonal antibody combinations must recognize separate epitopes on the antigen so they do not hinder each other’s binding and therefore be eligible as “matched pairs.” An example of a sandwich ELISA kit that detects circulating galactomannan is the *Platelia Aspergillus*, which due to the early detection of the antigen has been a major advance for managing patients at risk for invasive *aspergillosis* (Mennink-Kersten et al. 2004). For multiplexed sandwich assays there are several labeling and detection methods that can be employed (Wodicka et al. 1997; Scorilas et al. 2000; Moody et al. 2001; Wiese et al. 2001; Huang et al. 2001; Wang et al. 2002; Woodbury et al. 2002; Tam et al. 2002; Nielsen et al. 2003).

Matrix metalloproteinase-2 (MMP-2) and tissue inhibitor of metalloproteinases-2 (TIMP-2) levels in patients with systemic sclerosis were determined by means of sandwich ELISA in a study that concluded that serum TIMP-2 level can be used as a marker of the extent of skin sclerosis and disease activity added to the fact that TIMP-2 and MMP-2’s balance may play an important role in those patients (Yazawa et al. 2000). Moreover, another study used it to determine that increased pentosidine serum concentration in patients with osteoarthritis and its correlation with the cartilage destruction marker COMP (cartilage oligomeric matrix protein) in synovial fluid suggests that pentosidine may be important in osteoarthritis pathology and is a new potential osteoarthritis marker (Senolt et al. 2005).

#### 3.2.2 Western blot/immunoblot

Another immunological technique, Western blot or immunoblot, (Towbin et al. 1979; Burnette, 1981) is a method in biochemistry/molecular biology/immunogenetics to detect specific protein in a given sample of tissue homogenate or extract, while giving information about the size of that protein and how much protein has accumulated in cells. Proteins are electrophoresed into a gel (polyacrilamide gels, SDS-PAGE, are most common) to separate denatured proteins by size and charge and compared to a control, a “marker” or “ladder.” Proteins small in size migrate through the gel faster than larger ones. The separated proteins in an SDS-PAGE are then transferred out of the gel and onto a membrane, which is in general nitrocellulose or PolyVinylidine DiFluoride (a thermoplastic fluoropolymer) placed face-to-face with the gel by applying an electric current.

The detection of the membrane-blotted antigen requires: first, the blocking of the sites on the membrane that have no blotted protein from the gel, the addition and incubation of primary antibody with the membrane, which is a sensitive and specific detection tool, the addition of the secondary antibodies, which are anti-immunoglobulin antibodies coupled to a reporter group, such as to biotin, to alkaline phosphatase, e.g. goat anti-human IgG- alkaline phosphatase (Wallis et al. 1990), or to horseradish peroxidase, and directed against a species-specific portion of the primary antibody.

After excess second antibody is washed away from the blot, a substrate is added to the membrane and it will be converted by the reporter enzyme to a colored reaction product, this will cause the specific protein to be detected on the membrane to become visible, as in the case of utilizing monoclonal antibodies for SERCA 1 (clone IIH11) and SERCA 2 (Arruda et al. 2003). In case of a horse-radish peroxidase-linked secondary antibody, which is commonly used in combination with a chemiluminescent agent, the reaction product will produce fluorescence in proportion to the amount of specific protein bound. Placing a sensitive sheet of photographic film against the membrane will create an image of the antibodies bound to the blot upon its exposure to the light from the reaction. Another way of detection is by utilizing radioactive labeling (e.g. a radioactive isotope of iodine) rather than an enzyme coupled to the secondary antibody. This method is highly sensitive, but very rarely used as the other non-radioactive methods have more advantages of being cheaper, safer, and faster.

Western blot was recently used to confirm the presence of elevated serum heat shock protein 70 levels in the patients with sudden sensorineural hearing loss compared with normal controls. This study suggests that these levels of heat shock protein 70 might have a clinical role in predicting prognosis of hearing loss in the patients with sudden sensorineural hearing loss (Park et al. 2006). Also, Western blot was used to characterize Human Kallikrein 11, suggesting it as a candidate prostate and ovarian cancer biomarker, from seminal plasma (Luo et al. 2006). Moreover, it was utilized to validate that Heat-shock protein 27 is a potential biomarker for hepatocellular carcinoma and that it could help in the diagnosis of Hepatocellular carcinoma though further validation is needed (Feng et al. 2005).

### 3.3 Multi-dimensional Protein Identification Technology (MudPIT)

Multi-dimensional Protein Identification Technology (MudPIT) pioneered by Yates is an unbiased method for rapid and large-scale semi-quantitative proteome analysis and protein biomarker candidates identification (Wolters et al. 2001; Gao et al. 2005). MudPIT begins with multidimensional liquid chromatography, followed by tandem mass spectrometry, and finalizing by a database searching by the SEQUEST algorithm (Eng et al. 1994; Yates et al. 1995; Washburn et al. 2001), where SEQUEST is a database search program for performing protein identification and peptide sequencing by utilizing mass spectrometry fragmentation patterns to search protein and nucleotide databases (Tabb et al. 2001).

MudPIT is another approach which may help lessen many of the disadvantages associated with two-dimensional gel electrophoresis. The advantage of MudPIT using two chromatography steps interfaced back to back in a fused silica capillary is that the band broadening associated with many chromatographic steps, which can lead to resolution loss hence the running of components into one another, is avoided and also the capillary can be placed directly into the ion source of a mass spectrometer maximizing sensitivity. The mass spectrometer is a tandem electrospray, so peptides are ionized in the liquid phase, separated in a primary mass spectrometer, broken up using collision induced dissociation and analyzed again (Eng et al. 1994; Link et al. 1999; Gygi et al. 1999; Washburn et al. 2001; Han et al. 2001; Ideker et al. 2001; Tabb et al. 2002).

MudPIT was used to resolve and analyze, for example, the fractionated serum proteins which were trypsinized in an attempt to prove that several different prefractionation approaches should be used in parallel for a comprehensive identification of the serum proteins (Barnea et al. 2005). The Human Proteome Organization in another study used MudPIT to perform an extensive analysis of serum proteins in which they identified a large number of proteins (490) to further provide the foundation for future studies with clinically important disease states (Adkins et al. 2002). Analysis of the tandem mass spectra resulted in the identification of over 340 human serum proteins in a study that was trying to prove the efficacy of having Centrifugal ultrafiltration followed by MudPIT for the removal of large abundant proteins and the enrichment of the low molecular weight serum proteome (Tirumalai et al. 2003).

## 4. Biomarkers in Serum

### 4.1 Overview

Serum Prostate-Specific Antigen (PSA) and prostatic acid phosphatase are clinically established biomarkers in human serum. While PSA is used in insurance testing to assess the risk of underlying prostate cancer, other biomarkers are neither specific enough nor cost effective to use. Serum-protein biomarker approvals despite steady increases in the literature on potentially useful ones have seen a noticeable decrease. Before receiving US Food and Drug Administration (FDA) approval, biomarker candidates must undergo clinical validation. Yet that process is just beginning for most candidate markers. Moreover, among the FDA-approved biomarkers (see Table 2) only PSA is routinely used in standard clinical practice, and only two of the biomarkers have made it into the TNM staging guidelines, which is based on a combination of Tumor size or depth, lymph Node spread, and presence or absence of Metastases and provides the following information: a) a basis for prediction of survival, b) option of initial treatment, c) precise communication among healthcare providers, d) stratification of patients in clinical trials, e) and consistent reporting of the end result of cancer management. The ideal biomarker assay for staging should be specific, cost-effective, sensitive, fast, and durable against inter-operator and inter-institutional variability. It must also display clinical value afar that of the other types of information that are already available at the time of diagnosis (Ludwig and Weinstein, 2005). The following includes a discussion of the possible serum biomarkers that were found for cancerous and other major illnesses.

### 4.2 Cancer biomarkers

Cancer or tumor markers can be defined as tools that clinicians rely upon to answer clinically related questions concerning a cancer disease (Diamandis, 2002). A more preferred specific definition of a tumor marker is: a molecule, a process, or a substance that is altered quantitatively or qualitatively in precancerous or cancerous conditions, the alteration being detectable by an assay (Hayes et al. 1996). The tumor itself or the surrounding normal tissue as a response to tumor cells can produce those alterations (Hayes et al. 1996). The tumor marker itself can be mRNA, DNA, processes (apoptosis, angiogenesis, proliferation, etc.), or protein measured qualitatively or quantitatively by a suitable assay, which can be of various formats stretching from complex animal models to immunohistochemical test kits. The immunoassay, a well-characterized methodology, is probably the most commonly used format. In spite of this, there is a rapid progress in this field, and fresh and advanced assays, like microarrays and mass spectrometry, are becoming established technologies in tumor marker research (Schrohl et al. 2003). In the following paragraphs only biomarkers in “serum” will be discussed in both cancer and other disease cases.

#### 4.2.1 Prostate cancer biomarker

To date, predictive clinical markers in prostate cancer are the prostatic acid phosphatase (Gutman et al. 1936; Yam, 1974; Vihko, 1979; Lin et al. 1980; Sakai et al. 1992; Veeramani et al. 2005) and serum PSA (Sensabaugh, 1978; Cooper et al. 1990; Grignon and Hammond, 1995; Schrohl et al. 2003; Veeramani et al. 2005). Other markers have not earned sufficient support in the literature to be recommended for routine clinical application (Grignon and Hammond, 1995). Human prostatic acid phosphatase was used prior to the availability of PSA as a valuable surrogate marker for monitoring prostate cancer (Veeramani et al. 2005). Although PSA, the tumor-associated serum protein marker, is useful for monitoring prostate cancer progression, it is insufficient for diagnosing primary cancers or even early prostate cancer detection mainly due to its limited specificity at the desired sensitivity (Pannek and Partin, 1998, Djavan et al. 1999; Petricoin et al. 2002b; Adam et al. 2002; Qu et al. 2002; Wang et al. 2005; Engwegen Judith et al. 2006).

Identification of serum biomarkers for prostate cancer has been the aim of many studies. In one study, caspase-cleaved cytokeratin 18 (CK18-Asp396) serum marker was used to assess tumor apoptosis *in vivo* (Kramer et al. 2006). In another study, serum prostate secretory protein of 94 amino acids is able to help identify patients with high grade prostate cancer (Nam et al. 2006). In addition, serum hepatocyte growth factor activator was elevated in patients with advanced stage prostate cancer but further studies are needed to verify its clinical value (Nagakawa et al. 2005). Serum insulin-like growth factor binding protein-1 and -3 participate in an important role in regulation of prostate cancer cell growth. Results of studying serum insulin-like growth factor binding protein-3/PSA ratio suggested that it might be a useful prognostic marker in patients with advanced prostate cancer (Miyata et al. 2003). Moreover, an 80 kDa fragment of e-cadherin was believed to be a serum biomarker in a broad spectrum of prostate cancer cases (Kuefer et al. 2005). Today assays are available to detect a humoral immune response against alpha-methyl-acyl-CoA racemase, which may have the potential to complement PSA screening in identifying patients with clinically significant prostate cancer (Sreekumar et al. 2004).

#### 4.2.2 Breast cancer biomarker

Serum carcinoembryonic antigen (CEA), a glycoprotein normally found in embryonic endodermal epithelium, is elevated in 30–50% of patients with symptomatic metastatic breast cancer (Mughal et al. 1983; Williams et al. 1988; Robertson et al. 1991). MUC1 mucin, a glycoprotein, is expressed on the apical surface of epithelial cells under normal conditions. Only in case of tumors, the disruption of the normal tissue architecture due to the growing tumor allows MUC1 mucin to be shed into the circulation where it can be measured via immunoassays kits to detect cancer antigen 15.3 (CA 15.3), mucin-like carcinoma associated antigen, breast cancer mucin, and the like. Human kallikrein gene 5 was also found to be a potential biomarker in patients with ovarian and breast cancer (Yousef et al. 2003).

Currently, many biomarkers, particularly the hormonal and epidermal growth factor receptors, are utilized for breast cancer prognosis. Together with those predictive biomarkers (progesterone and estrogen receptors) (Schrohl et al. 2003), histological grade or Gleason system, axillary lymph node status, tumor size, and age, are currently used for selecting the suitable systemic therapy. Although none of the biomarkers in use have sufficient diagnostic, prognostic or predictive power across all categories and stages of breast cancer, more useful information can be generated if tumors are questioned with multiple markers with a right combination for each case, which is a challenging matter (Arciero et al. 2003; Somiari et al. 2005). In a recent study, one of the most popular serum biomarkers for the follow up of breast cancers and the early detection of breast cancer metastases is the cancer antigen Ca15.3, (Wang et al. 2005; Mathelin et al. 2006), a carbohydrate antigen commonly known as a “mucin,” which influences cell–cell interaction and cell growth (Banfi et al. 1997). In patients with early stage cancers, however, one study shows that Ca15.3 measurements are not helpful in diagnosis and the therapeutic decision making of patients with breast cancer (Lumachi et al. 2004). Her-2/neu, a cell membrane surface-bound tyrosine kinase, which is involved in the signal transduction pathways leading to cell growth and differentiation, and a glycoprotein MUC1 that can be recognized by CA27–29, a monoclonal antibody, are Food and Drug Administration (FDA) approved cancer biomarkers for monitoring breast cancer (Ludwig and Weinstein, 2005).

In a recent paper the clinical importance of serum pro-Iota collagen peptide and serum carboxyterminal telopeptide of type I collagen, CA15.3 and CEA was evaluated as compared to bone scintigraphy to detect metastatic breast cancer. It was concluded that combining serum CA15.3, carboxyterminal telopeptide of type I, and CEA would increase the sensitivity and specificity of the test, while pro-Iota collagen peptide alone or combined with carboxyterminal telopeptide of type I were not sensitive (Zissimopoulos et al. 2006).

#### 4.2.3 Ovarian cancer biomarker

Increased serum levels of CA125 (Whitehouse and Solomon, 2003), a mucin-type protein (Lloyd et al. 1997) that is often secreted into the blood by ovarian cells shed by cancer cells or also made by inflamed normal cells that line body parts, is widely used in the clinical setting to monitor ovarian cancer patients for early diagnosis of tumor recurrence (Bast et al. 1998) and follow-up of patients after treatment to calculate response (Rustin et al. 1999). However, it has low sensitivity and specificity and false-positive results can be obtained since CA125 is also produced in the ascites, a common factor present in both ovarian cancer and liver cirrhosis, and therefore the CA125 antigen alone can not be of diagnostic use in any patient with ascites or pleural effusions (Bergmann et al. 1986).

Many studies have been performed by different groups aiming to uncover serum biomarkers for ovarian cancer or even tentative ones for several stages of the cancer. One of which showed that isoforms of haptoglobin-1 precursor, a liver glycoprotein present in human serum can be used as a serum marker for ovarian cancer early detection (Ahmed et al. 2004). Determined by ELISA, two ovarian cancer biomarker proteins were discovered by another group and identified as hemoglobin-α and hemoglobin-β. But additional studies are required to further validate them as biomarkers (Ahn et al. 2005). In addition to those, some studies found that serum human kallikrein 6 (a serine protease) (Diamandis, 2002; Diamandis et al. 2003; Bayes et al. 2004; Oikonomopoulou et al. 2006) and human kallikrein 10 (Luo et al. 2001) concentrations seem to be new biomarkers for ovarian carcinoma and may have value for disease diagnosis and prognosis. Prostasin, a tryptic peptidase expressed in prostate, kidney, lung and airway (Tong et al. 2004), is overexpressed in epithelial ovarian cancer and should be investigated further as a screening or tumor marker, alone and in combination with CA125 (Mok et al. 2001). Osteopontin, a N-linked glycoprotein family of calcified extra-cellular matrix–associated protein, has been discussed as a potential diagnostic biomarker for ovarian cancer and showed evidence of an association between levels of osteopontin in serum and ovarian cancer suggesting that it would be worthwhile for future research assessing its clinical usefulness (Kim et al. 2002; Bramwell et al. 2006). Tumor-associated trypsin inhibitor’s elevated level is another important predictor of disease stage and future prognosis in ovarian cancer (Torre et al. 1991). Moreover, a study suggested that preoperative serum Human Chorionic Gonadotropin-beta is a strong independent prognostic factor in epithelial ovarian carcinoma (Higashida et al. 2001) when measured with a sensitive and specific method (Vartiainen et al. 2001). Furthermore, another study illustrates that Interleukin-8 could possibly act as a useful monitoring marker in patients with ovarian carcinoma (Mayerhofer et al. 2001). It was also found that an increased serum Carboxyterminal telopeptide of type I collagen concentration may reflect the spreading and aggressiveness of invasively growing ovarian cancer making serum Carboxyterminal telopeptide of type I collagen a clinically useful predictor of the clinical behavior of ovarian cancer (Santala et al. 2004).

#### 4.2.4 Lung cancer biomarker

People at high risk for lung cancer have been studied in an attempt to identify new biomarkers that may be predictive of precancerous lung lesions and their possible progression to lung cancer. A study showed that serum CEA level could be a predictive factor for the efficacy of gefitinib treatment (a drug that is used to treat non-small cell lung cancer also is being studied in the treatment of other types of cancer) while also considering CEA a prognostic factor for advanced non-small cell lung cancer patients undergoing this treatment (Okamoto et al. 2005). A more recent topic-related study revealed that increased pretreatment serum macrophage colony-stimulating factor level is a significant independent predictor of poor survival in patients with non-small cell lung cancer, where CEA levels were shown but couldn’t prove to be an independent prognostic factor as macrophage colony-stimulating factor did (Kaminska et al. 2006). Currently, there are no satisfactory biomarkers available to screen for lung cancer.

#### 4.2.5 Other cancer biomarkers

In order to improve the prognosis of cancer patients, it is crucial to explore serum biomarkers for its early diagnosis. One of the serum biomarkers is the alpha-fetoprotein for tumors of the liver (Johnson et al. 2000; Johnson, 2001), non seminomatous testis, and other germ cell line tumors. Human corionic gonadotropin-β, a glycoprotein, elevated serum level indicates the presence of metastatic testicular seminoma tumors but lactic acid dehydrogenase serum level, rather than human corionic gonadotropin-β, is more useful as a prognostic indicator for those patients with seminoma (Hori et al. 1997). Even though serum calcitonin and CEA are biomarkers for medullary thyroid carcinoma (Hamada et al. 1976), some recent studies report that those biomarkers are of no clinical use (Bockhorn et al. 2004), while other studies add progastrin-releasing peptide, which is considered to be a specific marker for small cell lung carcinoma, as an additional marker for the diagnosis and monitoring the response to therapy in patients with medullary thyroid carcinoma (Ide et al. 2001). The evaluation of the concentration of metalloproteinase-2 and its tissue inhibitor TIMP-2 in peripheral blood serum may be useful for the differentiation between benign and malignant thyroid tumors, but their concentrations in patients with thyroid cancer did not significantly correlate with the clinical staging of thyroid cancer (Pasieka et al. 2004). Measuring the levels of thyroglobulin, a protein produced by the thyroid gland, in the blood have been approved by the FDA as a monitoring biomarker for thyroid cancer (Ludwig and Weinstein, 2005). In systemic malignant melanoma, melanoma-inhibiting activity was found to represent a serum marker for this cancer showing a high sensitivity and specificity (Bosserhoff et al. 1997). A studying group have discussed human chronic gonadotropin-beta as being most commonly elevated (>10 mIU/ml) in the serum of gynecological cancers especially ovarian cancer (Higashida et al. 2001), but it is also elevated in colorectal (Lundin et al. 2000), pancreas (Syrigos et al. 1998), and kidney (Torre et al. 1991), cancers and, therefore, can be referred to as a “current serum cancer marker.” D-dimer’s elevated levels in serum, a fibrin degradation product, has also been proposed as an important prognostic biomarker for metastatic colorectal carcinoma (Blackwell et al. 2004) but its use is limited by its low specificity. CEA is reported as a colon cancer marker having low specificity and insufficient sensitivity to be used as a screening marker, as it can be elevated by many other factors than cancer; smoking for instance raises CEA levels, but can be helpful in follow-up (Bast et al. 1996; Chatterjee and Zetter, 2005). CA19–9, a serum-derived single carbohydrate, has been approved by FDA as a cancer biomarker for monitoring pancreatic cancer (Ludwig and Weinstein, 2005). Comparing serum lipoprotein in patients with cancer to non-cancer subjects revealed an association of cancer with low serum total cholesterol and a characteristic of low low-density lipoprotein-cholesterol, low high-density lipoprotein-cholesterol and relatively high serum triglycerides, where the abnormality is a common feature of both hematological and solid tumors and is not entirely explained by poor nutrition (Fiorenza et al. 2000). Polanski and Anderson (2006) have done comprehensive literature review and compiled a list of 1261 proteins believed to be differentially expressed in human cancer. Among them, only nine have been approved as “tumor associated antigens” by the FDA, i.e. CEA, Her-2/neu, bladder tumor antigen, thyroglobulin, alpha-fetoprotein, PSA, CA125, CA19.9, CA15.3. Only a small fraction of proteins are candidate plasma biomarkers that could be further evaluated and validated for future early cancer detection, prognosis, and monitoring efficacy of specific treatment options.

### 4.3 Biomarkers for other diseases

#### 4.3.1 Heart disease

B-type natriuretic peptide, nesiritide, was reported as a prognostic marker in acute coronary syndromes (McCullough et al. 2002). Several likely uses are now considered for B-type natriuretic peptide and its terminal prohormone fragment, N-terminal proB-type natriuretic peptide, in adults: (I) to examine heart failure therapy’s efficiency (Troughton et al. 2000; Rodeheffer, 2004); (II); to predict prognosis (Anand et al. 2003; Maisel et al. 2004); (III) to check asymptomatic persons at threat for asymptomatic ventricular dysfunction (Nakamura et al. 2002; Redfield et al. 2004); and (IV) to recognize the failure of the heart of patients having dyspnea or the like symptoms suggesting heart disease (McCullough et al. 2002; Maisel et al. 2002). Although, there are available commercial kits for B-type natriuretic peptide and N-terminal pro-B-type natriuretic peptide, which makes them candidate markers for heart disease in daily practice (Nasser et al. 2005), standardization of these assays is deficient, diverse cutoff values have been reported, and the effect of age, gender (Redfield et al. 2002), and possibly renal function, on normal values (McCullough Peter and Sandberg Keisha, 2003) may complicate the use of B-type natriuretic peptide as a marker for heart disease. A recent study evaluating serum Levels of N-terminal proB-type natriuretic peptide reveal it as an early cardiac marker of carbon monoxide poisoning (Davutoglu et al. 2006) and of heart toxicity caused by amyloidogenic light chains (Palladini et al. 2003). Another study assessing serum N-terminal pro-B-type natriuretic peptide and cardiac troponin T levels and echocardiography was recently performed and concluded that their simultaneous measurement allows for precise acute pulmonary embolism prognosis (Kostrubiec et al. 2005). A scientific group hunted to describe the relationship between cholesterol and survival in patients with chronic heart failure and concluded that lower serum total cholesterol is independently associated with a worse prognosis (Rauchhaus et al. 2003).

Concentrations of C-reactive protein (CRP), serum amyloid A, and interleukin-6 were increased in patients with coronary heart disease but failed to correlate with acuteness of coronary disease. Therefore, the study concluded that these markers might reflect the diffuse atherosclerotic process in the vascular system rather than the degree of localized obstruction from coronary lesions (Rifai et al. 1999). A later study concluded that high-sensitivity CRP has a prognostic utility in patients with acute coronary syndromes, and it is also considered a strong independent predictor of future coronary events in seemingly healthy subjects (Rifai and Ridker, 2001). Recently, a study verified that the first paroxysmal episode of lone atrial fibrillation is associated with elevated high-sensitivity CRP levels, proposing that high-sensitivity CRP may be a marker for inflammatory states that may prop up the initiation of lone atrial fibrillation (Hatzinikolaou-Kotsakou et al. 2006).

#### 4.3.2 Rheumatoid Arthritis (RA)

Persistently active rheumatoid arthritis (RA), in most patients, has no early biomarker, and this leads to high possibility of joint destruction and disability (Liao et al. 2004). Although, available biomarkers, like CRP or erythrocyte sedimentation rate, offer good correspondence parallel with disease activity, yet they do not predict subsequent severity (Riel et al. 1998). The evaluation of the level of stromelysin-1 (matrix metalloproteinase-3) in serum presents a particularly useful marker of inflammatory activity in the joints of patients with RA (Yoshihara et al. 1995; Yamanaka et al. 2000). In addition to melanoma-inhibiting activity’s role in being a serum marker for progression of malignant melanoma (see 4.2.5), a study revealed that it might also be useful in the differential diagnosis of RA *vs.* non-destructive rheumatic diseases via checking for the presence of elevated levels of melanoma-inhibiting activity in serum, which correspondingly means a very likely joint destruction in RA (Muller-Ladner et al. 1999).

Levels of a marker for aggrecan turnover in cartilage (CS846-epitope), which is normally found in chondroitin sulfate of the cartilage proteoglycan aggrecan, are found to be elevated in serum in chronic RA, even though the levels are found to be depressed in rapid progressive RA (Mansson et al. 1995). Some studies showed that a neoepitope marker for degradation of type II collagen in cartilage, which was generated by collagenases, is increased in the serum and urine of patients with RA (Poole et al. 2004) making type II collagen in cartilage a specific marker for cleavage of type II collagen (Verstappen et al. 2006). Recent studies show that interleukin-15 may be used as a marker for the evaluation of severity of juvenile rheumatoid arthritis (Cao et al. 2006). Another study’s evaluation of the degree of clinical rheumatoid arthritis activity based on the concentrations of cytokines tumor necrosis factor-α, interleukins -12, -15, and -18 in serum and synovial fluid concluded that cytokines concentrations could be good indicators of the degree of the general activity of RA (Petrovic-Rackov, 2006). The investigation of matrix metalloproteinases-2, -9, inhibitors of matrix metalloproteinase-1, and -2 levels in serum and synovial fluid in patients with RA and psoriatic arthritis deduced that the evaluation of synovial fluid concentrations is more reliable than that determined in serum (Giannelli et al. 2004). The vascular endothelial growth factor can also be detected in serum of patients with RA (Kikuchi et al. 1998; Harada et al. 1998), where vascular endothelial growth factor level is related to RA disease activity, therefore, suggesting that vascular endothelial growth factor may play some role in the pathogenesis of RA (Harada et al. 1998; Sone et al. 2001). Auto-antibodies to α-enolase, an enzyme of the glycolytic pathway, were found to be present in the sera of patients in very early RA cases, therefore, having potential diagnostic and prognostic value for RA (Saulot et al. 2002).

In a recent paper comparing marker proteins in serum and synovial fluid in patients with advanced osteoarthrosis (OA) and RA deduced that the only marker protein that revealed distinct higher levels in the serum than in the synovial fluid was matrix metalloproteinase-13, but further investigations may provide more information about the value of matrix metalloproteinase-13 as a potential marker to monitor the course of RA and OA (Andereya et al. 2006). Moreover, in another recent study myeloid-related protein 8 was confirmed by MS/MS to be present in serum of patients with erosive RA (Liao et al. 2004). Additionally, mass spectrometry identified protein biomarkers of disease severity in the synovial fluid and serum of patients with rheumatoid arthritis. Those biomarkers are high levels of CRP, S100A8/calgranulin A, S100A9/calgranulin B, and S100A12/calgranulin C proteins. Those proteins were elevated in the serum of patients with erosive disease compared to patients with nonerosive RA or healthy individuals (Liao et al. 2004).

An investigation of whether increased cartilage oligomeric matrix protein in serum is a specific marker for joint destruction done by comparing serum cartilage oligomeric matrix protein between patients with RA and patients with other inflammatory rheumatic diseases with less cartilage-destructive arthritis was able to confirm the conclusion that serum cartilage oligomeric matrix protein levels are highly specific markers for the cartilage degradation process in RA (Skoumal et al. 2004).

#### 4.3.3 Asthma biomarker

Neutrophil-mediated inflammatory events have been shown to be involved in exacerbation of childhood asthma and that the screening of urinary-trypsin-inhibitor-serum concentrations might be useful for evaluating the neutrophil-mediated inflammation in childhood asthma attack (Yasui et al. 2003). Serum esinophil cationic protein may also be used to predict a response to corticosteroid therapy in adult patients with asthma (Sorkness et al. 2002). Serum tryptase detected with B12 monoclonal antibody-based immunofluoroassay has been suggested as a marker of allergic airway inflammation in asthma (Taira et al. 2002).

#### 4.3.4 Cystic fibrosis

In Cystic Fibrosis (CF) patients, an elevated level of serum CA 19–9 (a tumor-associated antigen) concentration have been observed in several studies (Duffy et al. 1985; Buamah et al. 1986; Roberts et al. 1986). Trypsinogen has been reported to be an excellent screening test for CF in young infants (Cleghorn et al. 1985). As there is no straightforward correlation between CF-transmembrane conductance regulator genotype and CF lung disease severity, finding of novel therapeutic strategies that can efficiently overcome this pathology isn’t easy (Kerem et al. 1990). In a recent study the elevation of tissue inhibitor of metalloprotein-ases-1 (TIMP-1), collagen-IV, prolyl hydroxylase serum concentrations suggest that these may be indicators of hepatic fibrogenesis in cystic fibrosis (Pereira et al. 2004).

## Conclusions

Human serum is extremely complex thus is the most difficult protein-containing sample to characterize. This review examines the various proteomic techniques utilized for each step in the process of human serum sample preparation or treatment, for its prefractionation or separation, and for the analysis to identify the corresponding serum biomarkers related to a specific disease or during different stages of a disease development. The discussed proteomic techniques are utilized for identifying protein-based serum biomarkers. The importance of biomarker identification cannot be underestimated. A fast growing area of basic, translational, and clinical research interest is the applications of proteomics techniques to preventive, diagnostic, and prognostic medicine. Protein biomarkers could facilitate the early detection of the onset of a disease at a curable stage and identification of subgroup of patients who respond well to certain types of drug treatment from those who do not. The knowledge generated may provide biochemical indicators for predicting patients’ risk for cancer, heart attack, or other diseases and their different responses to the same therapy for optimal patient care and management.

Although many serum biomarkers were discussed, only a few are FDA approved for disease diagnosis and prognosis, including cancer biomarkers in human serum, CEA, Her-2/neu, bladder tumor antigen, thyroglobulin, alpha-fetoprotein, PSA, CA125, CA19.9, CA15.3, and some other cancer biomarkers, as well as cardiovascular disease biomarkers Troponin I and B-type natriuretic peptide (for more detailed review, see Polanski and Anderson, 2006). Many biomarkers remain to be validated for accurate clinical identification of a particular disease. It is more desirable to combine different techniques and identify a panel of biomarkers to accurately measure the disease biomarker levels and establish criteria for disease diagnosis and prognosis. Moreover, many techniques still lack the proper sensitivity, specificity, and reproducibility for the identification of serum or other biomarkers. ELISA is a quantitative assay based on specific antibody-antigen binding and is commonly used to analyze biomarkers due to its sensitivity, specificity, and simplicity. Unfortunately, specific antibodies against many biomarkers are not available. In addition, due to the low abundance of some biomarkers, those biomarkers are masked by other more abundant proteins, and thus, difficult to be detected. To enhance future serum biomarker identification, techniques should be improved and combinations of different technologies and statistical analysis are required to increase the sensitivity, reproducibility, and specificity of biomarker detection.

## Figures and Tables

**Table 1:** Advantages and Disadvantages of Albumin Elimination *versus* Serum Fractionation.

Techniques		Advantages	Disadvantages
**Albumin Elimination Techniques**	Cibacron Blue F3GA	- Fast	- Loss of proteins bound to albumin
	Antibody-Based Albumin Elimination	- Does not require specialized instrumentation	
**Serum Fractionation Techniques**	Anion Exchange Chromatography	- High Recovery Rate	
	Reversed-Phased High Performance Liquid Chromatography	- Can be coupled with other separation techniques	- Requires specialized Instrumentation
	Size Exclusion Chromatography	- Can be optimized to fractionate the serum sample into many fractions each enriched with target proteins	- Time consuming

**Table 2:** FDA-approved and Potential Disease-serum Biomarkers.

Disease	FDA approved Biomarkers	Potential Biomarkers
Prostate	PSA	Prostatic acid phosphatase, CK18-Asp396, prostate secretory protein of 94 amino acids, hepatocyte growth factor activator, insulin-like growth factor binding protein-1 and -3, E-cadherin, and alpha-methylacyl-CoA racemase.
Breast	CA15.3, Her-2/neu, and CA27–29	CEA, Human kallikrein genes 5, and serum carboxyterminal telopeptide of type I collagen.
Ovarian	CA125	Isoforms of haptoglobin-1 precursor, hemoglobin-α, hemoglobin-β, human kallikrein 6 & 10, Prostasin, Osteopontin, Tumor-associated trypsin inhibitor, preoperative serum Human Chorionic Gonadotropin-beta, Interleukin-8, and serum Carboxy-terminal telopeptide of type I collagen.
Lung (non-small cell lung cancer)	none	CEA and pretreatment serum macrophage colony-stimulating factor.
Lung (small cell lung cancer)	none	Pro-gastrin-releasing peptide
Liver	none	Alpha-fetoprotein
Non Seminomatous	alpha- fetoprotein	
Testicular Seminoma	Human corionic gonadotropin-β	
Thyroid	Thyroglobulin	MMP-2 and TIMP-2.
Medullary Thyroid	none	Calcitonin, CEA, and pro-gastrin-releasing peptide.
Melanoma	none	Melanoma-inhibiting activity
Colorectal	none	Human chronic gonadotropin-beta and D-dimer.
Pancreas	CA19–9	Human chronic gonadotropin-beta
Kidney	none	Human chronic gonadotropin-beta
Colon	none	CEA
Heart Disease	none	B-type natriuretic peptide, N-terminal pro-B-type natriuretic peptide, cardiac troponin T, and high-sensitivity CRP.
Rheumatoid Arthritis	none	CRP, erythrocyte sedimentation rate, stromelysin-1 (MMP-3), melanoma-inhibiting activity, CS846-epitope, neoepitope marker for degradation of type II collagen in cartilage, interleukin-15, cytokines, vascular endothelial growth factor, myeloid-related protein 8, CRP, S100A8/calgranulin A, S100A9/calgranulin B, S100A12/calgranulin C proteins, and serum cartilage oligomeric matrix protein.
Asthma	none	Serum urinary-trypsin-inhibitor, serum esinophil cationic protein, and serum tryptase.
Cystic Fibrosis	none	CA 19–9, Trypsinogen, TIMP-1, collagen-IV, and prolyl hydroxylase.

## Abbreviations

2-DE: two-dimensional gel electrophoresis
2DLC-MS: two-dimensional liquid chromatography mass spectrometry
CA 15.3: cancer antigen 15.3
CA 19–9: cancer antigen 19–9, a tumor-associated antigen
CA125: cancer antigen 125, a mucin-like protein
CEA: carcinoembryonic antigen
CF: Cystic Fibrosis
CRP: C-reactive protein
ELISA: enzyme-linked immunosorbent assay
ESI-MS/MS: electrospray ionization tandem mass spectrometry
FDA: Food and Drug Administration
IPG: immobilized pH gradient
MALDI-TOF-MS: matrix-assisted laser desorption-ionization time-of-flight mass spectrometry
MDLC: multidimensional liquid chromatography
MUC: mucin
MudPIT: multi-dimensional protein identification technology
OA: osteoarthrosis
pI: isoelectric point
PSA: prostate-specific antigen
RA: rheumatoid arthritis
RP-HPLC: reversed-phased-high performance liquid chromatography
RPLC: reversed phase liquid chromatography
SDS-PAGE: sodium dodecyl sulfate polyacrylamide gel electrophoresis
SEC: size exclusion chromatography
SELDI: surface-enhanced laser desorption ionization
SELDI-TOF: surface-enhanced laser desorption ionization-time of flight
